# Nucleotide-bound crystal structures of the SARS-CoV-2 helicase NSP13

**DOI:** 10.1107/S2053230X25005266

**Published:** 2025-07-10

**Authors:** Patrick Kloskowski, Piotr Neumann, Annette Berndt, Ralf Ficner

**Affiliations:** a Department of Molecular Structural Biology, Institute of Microbiology and Genetics, Göttingen Center of Molecular Biosciences (GZMB) University of Göttingen Justus-von-Liebig-Weg 11 37077Göttingen Germany; b Cluster of Excellence ‘Multiscale Bioimaging: From Molecular Machines to Networks of Excitable Cells’ (MBExC) University of Göttingen Justus-von-Liebig-Weg 11 37077Göttingen Germany; University of Leipzig, Germany

**Keywords:** SARS-CoV-2, NSP13 helicase, nucleotide-binding sites, inorganic phosphate, ADP-bound structure, ATP-bound structure, COVID-19

## Abstract

Nucleotide-bound crystal structures of SARS-CoV-2 NSP13 in ADP- and ATP-bound states were resolved to 1.8 and 1.9 Å, respectively. The ADP-bound model captures a state immediately following ATP hydrolysis, with both ADP and orthophosphate still present in the active site. Further comparative analysis revealed that crystal packing influences NSP13 by stabilizing the nucleotide-binding site, underscoring the importance of accounting for these effects in structure-based drug design targeting NSP13.

## Abstract 2

Nucleotide-bound crystal structures of SARS-CoV-2 NSP13 capture a state immediately following ATP hydrolysis and reveal how crystal packing can affect structure-based drug design targeting NSP13.

## 1. Introduction

Nonstructural protein 13 (NSP13), a helicase, is a critical component of the replication machinery in SARS-CoV-2. During the viral life cycle, NSP13 associates with other non­structural proteins to form the replication–transcription complex (RTC; Chen *et al.*, 2020 ▸). NSP13 exhibits diverse enzymatic activities, including NTPase activity, NTP-dependent translocation on RNA and DNA, and RNA 5′-triphos­phatase activity (Newman *et al.*, 2021 ▸), which enable it to perform essential roles in the replication and transcription of the SARS-CoV-2 genome. In particular, NSP13 contributes to resolving RNA secondary structures, displacing nucleic acid-bound proteins, facilitating transcriptional backtracking, regulating replication fidelity, enabling mRNA capping and mediating template switching (Grimes & Denison, 2024 ▸). The critical role of NSP13 in viral replication is further emphasized by its high conservation across coronaviruses, including SARS-CoV-1 and MERS-CoV. Remarkably, NSP13 from SARS-CoV-2 differs by only a single amino-acid substitution (V570I) from its SARS-CoV-1 counterpart and shares 70% sequence identity with MERS-CoV NSP13. Furthermore, structural comparisons using the *DALI* server (Holm *et al.*, 2023 ▸; http://ekhidna2.biocenter.helsinki.fi/dali/) revealed significant structural homology among these NSP13 variants, as shown by high *DALIZ*-scores (≥42.1 for PDB entries 6szl, 6jyt and 5wwp).

**Figure 1:**
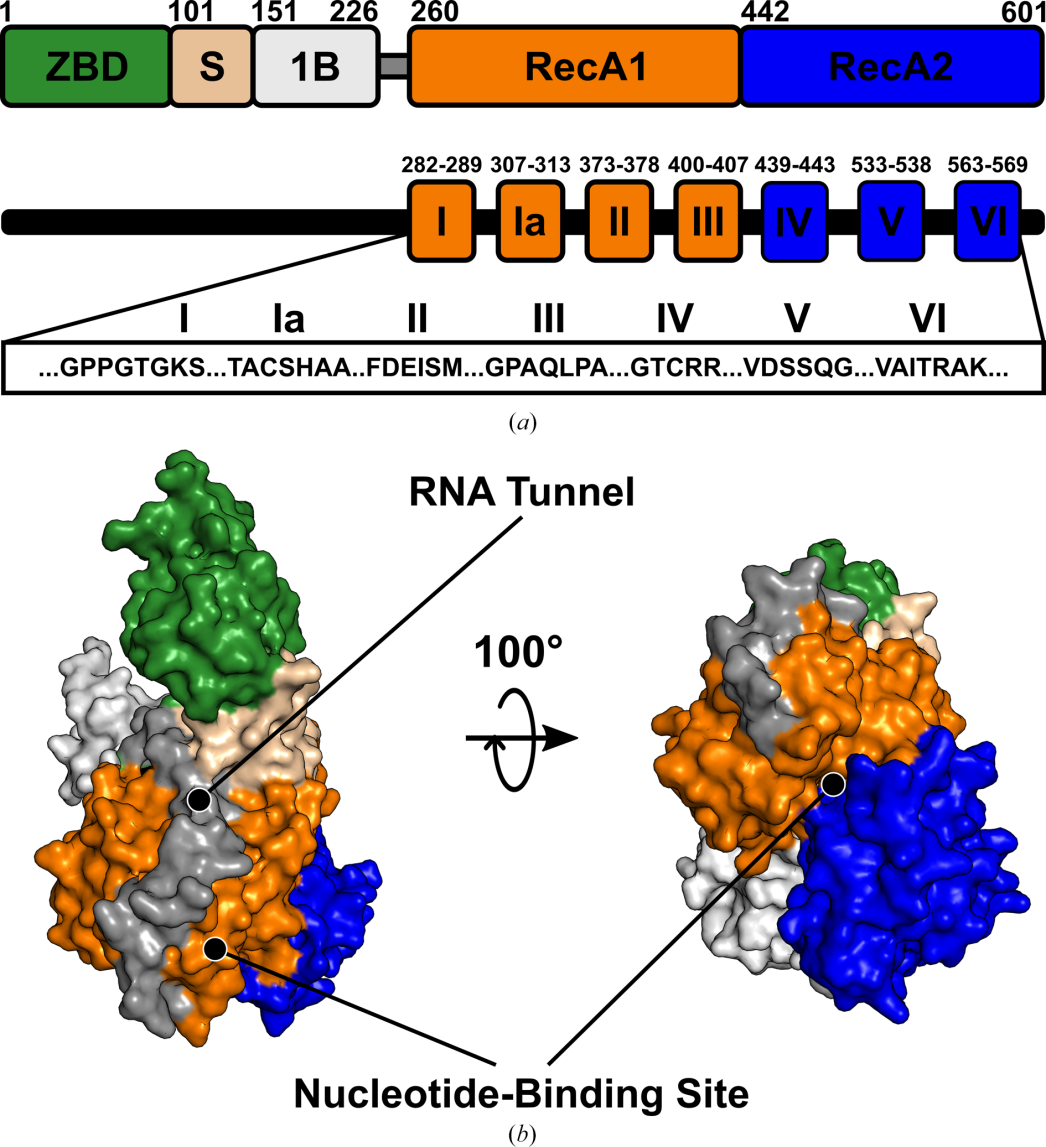
Domain architecture and structural features of SARS-CoV-2 NSP13. (*a*) Schematic representation of the domain organization of NSP13, consisting of five domains: the zinc-binding domain (ZBD, green), stalk domain (S, wheat), 1B domain (grey), RecA1 domain (orange) and RecA2 domain (blue). The 1B domain and RecA1 domain are connected by an unstructured linker (dark grey). The conserved helicase motifs (I–VI) within the RecA1 and RecA2 domains are displayed with their respective residue ranges and sequences within NSP13. (*b*) Surface representation of the three-dimensional structure of NSP13, with domains coloured as in (*a*). Key structural features include the nucleotide-binding site, formed between the RecA1 and RecA2 domains, and the RNA tunnel, formed by all domains except the ZBD. The structure is presented in two orientations rotated horizontally by 100°.

Known crystal and cryo-electron microscopy (cryo-EM) structures of SARS-CoV-2 NSP13 reveal a 67 kDa monomer with a triangular pyramidal architecture, consisting of five distinct domains: an N-terminal zinc-binding domain (ZBD), a stalk domain, the 1B domain and two RecA-like helicase core domains (RecA1 and RecA2), as illustrated in Fig. 1 ▸. The ZBD interacts with NSP8, as observed in cryo-EM structures, anchoring NSP13 to the RTC and facilitating its integration into replication and transcription processes (Chen *et al.*, 2020 ▸). The remaining domains, including the 1B, stalk and RecA domains, form the RNA-binding tunnel, while the RecA1 and RecA2 domains also form the nucleotide-binding site of NSP13. Interestingly, the nucleotide-binding site shows significant structural similarity to the human Upf1 helicase (hUpf1; PDB entry 2gk7; *DALIZ*-score 22.7), classifying NSP13 as a Upf1-like helicase within superfamily 1B (SF1B). The SF1B helicases are characterized by their 5′–3′ polarity, their ability to act on both DNA and RNA substrates, and conserved motifs within their RecA domains (Raney *et al.*, 2013 ▸).

The helicase activity of NSP13 is facilitated by a series of conserved motifs, as described by Fairman-Williams *et al.* (2010 ▸). These motifs include motif I (residues 282–289, GPPGTGKS), motif Ia (residues 307–313, TACSHAA), motif II (residues 373–378, FDEISM), motif III (residues 400–407, GDPAQLPA) and motif IV (residues 439–443, GTCRR; sometimes referred to as motif IIIa in the SF1 context), as well as motif V (residues 533–538, VDSSQG) and motif VI (residues 563–569, VAITRAK). Motifs I, II and IV play key roles in ATP binding and hydrolysis, whereas motifs Ia, III, V and VI are involved in RNA binding and in coupling the energy from ATP hydrolysis to helicase activity (Raney *et al.*, 2013 ▸).

Interestingly, the nucleotide-binding site of NSP13 is highly conserved across coronaviruses (Newman *et al.*, 2021 ▸). An analysis of the amino acids lining this site across 27 α- and β-coronaviruses revealed that 79% of the residues are identical, underscoring the functional importance of this site and establishing it as an attractive target for antiviral drug development. Detailed structural analyses have provided valuable insights into the nucleotide-binding site, highlighting its interactions and viability as a drug target. Remarkably, the AMP-PNP-bound crystal structure revealed distinct nucleotide-binding modes, while a fragment screen identified several fragments that bind to this site, offering promising starting points for inhibitor development (Newman *et al.*, 2021 ▸). Additionally, cryo-EM structures have illuminated the role of NSP13 within the RTC, showing NSP13 bound to RNA and to the ADP–Mg^2+^–AlF_3_ complex, which mimics the transition from ATP to ADP (Chen *et al.*, 2022 ▸).

In this study, nucleotide-bound crystal structures of SARS-CoV-2 NSP13 are presented, featuring bound ADP and ATP. The ADP-bound structure captures the product state of NSP13, offering valuable structural insights following the hydrolysis of ATP. Furthermore, the findings highlight how crystal packing may influence the observed NSP13 conformation, revealing challenges in accurately resolving ligand and inhibitor complexes with SARS-CoV-2 NSP13.

## 2. Methods

### 2.1. Protein production and purification

The production and purification of NSP13 from SARS-CoV-2 (NCBI Accession YP_009725308) were performed as described in Kloskowski *et al.* (2025 ▸). Briefly, a modified pET-52b(+) vector was used to express NSP13 with an N-terminal Strep-tag II in *Escherichia coli* Rosetta 2 (DE3). The protein was purified using a StrepTrap XT column (Cytiva), followed by tag removal via TEV protease digestion and further purification by size-exclusion chromatography on a Superdex 200 16/60 column (Cytiva). The purified protein was dissolved in a buffer consisting of 50 m*M* HEPES pH 7.5, 500 m*M* NaCl, 0.5 m*M* TCEP and then concentrated to 20 mg ml^−1^.

### 2.2. Protein crystallization, ligand co-crystallization and ligand soaking

The protein crystals used for soaking and co-crystallization experiments were obtained as described in Kloskowski *et al.* (2025 ▸) and in Table 1 ▸. Briefly, the reservoir solution comprised 16% ethylene glycol, 8% PEG 8000, 0.05 *M* HEPES, 0.05 *M* MOPS, 0.03 *M* sodium nitrate, 0.03 *M* sodium phosphate, 0.03 *M* ammonium sulfate and 9% MPD.

For co-crystallization, NSP13 was incubated with ADP or ATP at a fivefold to 20-fold molar excess for 30–60 min at room temperature (RT) before crystallization. In soaking experiments, pre-grown crystals were exposed to 5–50 m*M* ADP or ATP for 30 min to 2 h. Prior to flash-cooling, crystals were cryoprotected in reservoir solution supplemented with increased concentrations of ethylene glycol and PEG 8000 (Table 1 ▸). No magnesium ions were included in any soaking or co-crystallization solution, as their presence would stimulate ATP hydrolysis. ADP was soaked under the same metal-free conditions to ensure that the ATP and ADP data sets were collected in an identical chemical environment.

### 2.3. Data collection, structure determination and ligand identification

Diffraction data from putative nucleotide-bound SARS-CoV-2 NSP13 crystals were collected on EMBL beamline P13, PETRA III, DESY, Hamburg, Germany and processed with *autoPROC* (Vonrhein *et al.*, 2011 ▸), which integrates *XDS*, *POINTLESS*, *AIMLESS* and *CCP*4 (Evans & Murshudov, 2013 ▸; Kabsch, 2010 ▸; Winn *et al.*, 2011 ▸; Evans, 2006 ▸). Data-collection and processing statistics are provided in Table 2 ▸. Structure determination was performed with *DIMPLE*(Wojdyr *et al.*, 2013 ▸), a macromolecular crystallography pipeline utilizing programs from the *CCP*4 suite (Murshudov *et al.*, 2011 ▸; Agirre *et al.*, 2023 ▸). PDB entry 6zsl (Newman *et al.*, 2021 ▸) was used as the initial search model for molecular replacement.

*DIMPLE*’s integrated blob function reported unmodelled electron density at the nucleotide-binding site of the soaked NSP13 crystals located in each of the two monomers of the asymmetric unit (chains *A* and *B*). It was attributed to ADP or ATP, depending on the soaking experiment. In chain *B* of both structures, the difference electron-density map indicated the presence of a partially occupied β-phosphate that was bound prior to soaking experiments. The refined occupancies amounted to 0.30 and 0.29 for the ADP- and ATP-bound structures, respectively. Near each bound nucleotide molecule, an inorganic phosphate originating from the crystallization conditions (Table 1 ▸) was observed. Additionally, an extra ADP or ATP molecule was identified between the two NSP13 chains occupying the asymmetric unit. Further unmodelled electron density corresponding to the reservoir buffer component MOPS was observed at the 5′-RNA binding site of chain *A* and chain *B* in each crystal structure, consistent with previous findings (Kloskowski *et al.*, 2025 ▸).

Each atomic model underwent manual rebuilding in *Coot* (Emsley *et al.*, 2010 ▸) with alternating reciprocal-space and real-space refinement cycles using a *Phenix*-based refinement pipeline (Garbers *et al.*, 2024 ▸). Refined structures of ADP- and ATP-bound NSP13 have been submitted to the Protein Data Bank as PDB entries 9i51 and 9i53, respectively (Table 3 ▸). Figures were prepared using the open-source version of *PyMOL* (version 2.6; Schrödinger).

### 2.4. Interaction analysis

Intermolecular interactions between the residues of SARS-CoV-2 NSP13, the nucleotides ADP or ATP and the inorganic phosphate were analysed using the *Arpeggio* webserver (https://biosig.lab.uq.edu.au/arpeggioweb/; Jubb *et al.*, 2017 ▸). This analysis provided detailed insights into the interactions at the nucleotide-binding site, highlighting both similarities and differences between the ADP-bound and ATP-bound structures of SARS-CoV-2 NSP13.

## 3. Results

Nucleotide-bound crystal structures of SARS-CoV-2 NSP13 were determined in ADP-bound and ATP-bound states at resolutions of 1.8 and 1.9 Å, respectively (Table 2 ▸). NSP13, a 67 kDa protein with a triangular pyramidal architecture, comprises five distinct domains, including two RecA-like domains that form its nucleotide-binding site (Fig. 1 ▸). Considering its critical role in viral RNA replication and its high conservation, NSP13 represents a compelling target for antiviral development against SARS-CoV-2 and other coronaviruses with pandemic potential.

**Figure 2:**
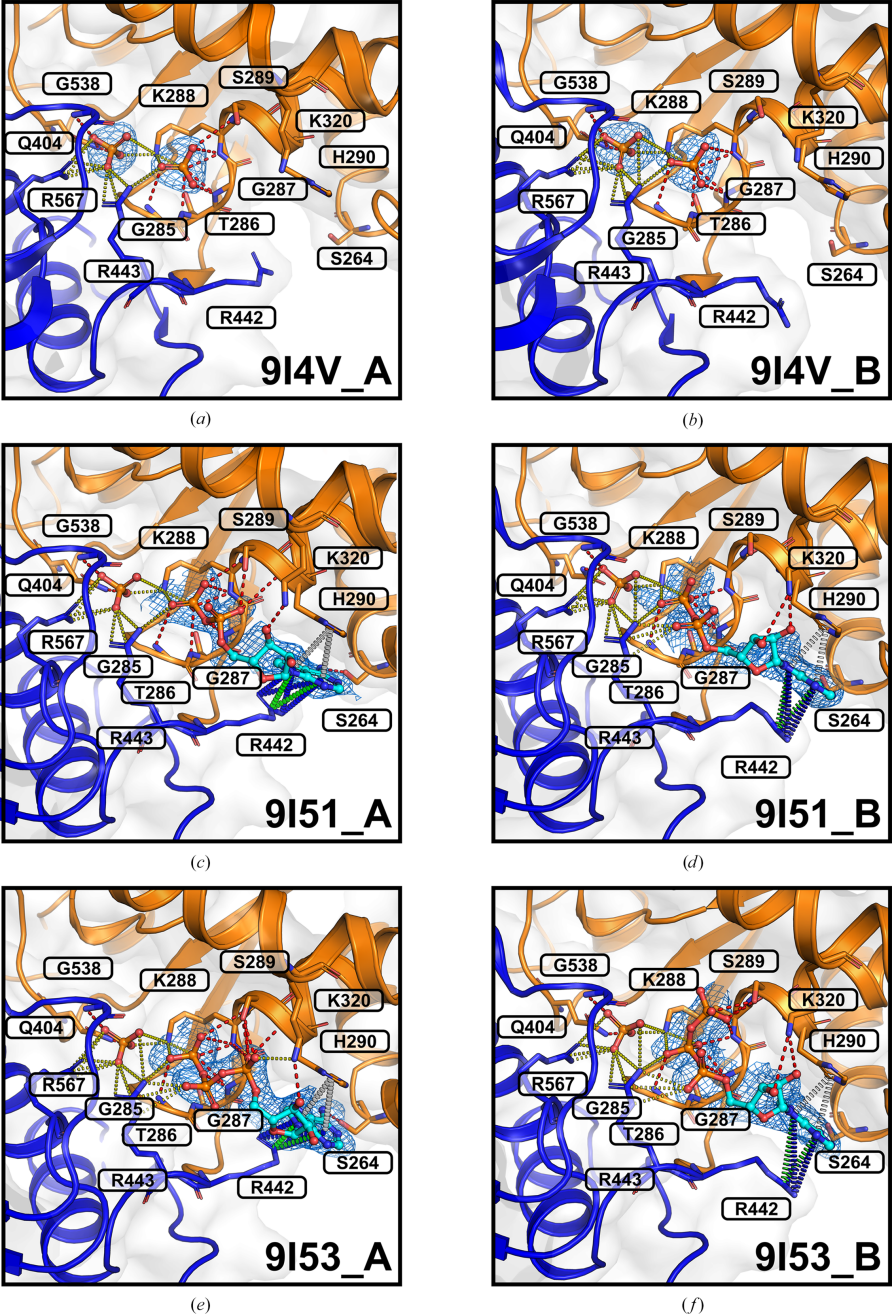
Phosphate and nucleotide binding in the phosphate-bound, ADP-bound and ATP-bound structures of NSP13. NSP13 is depicted in cartoon representation, showing the RecA1 domain (orange) and the RecA2 domain (blue). Phosphates are shown in ball-and-stick format coloured by atom, and the nucleotide is shown in cyan ball-and-stick format. Interacting residues are labelled and shown as sticks, with hydrogen bonds (red), salt bridges (yellow), π–π interactions (white), cation–π interactions (green) and donor–π interactions (blue) depicted. The polder OMIT *mF*_o_ − *DF*_c_ electron-density map for inorganic phosphate, ADP and ATP is displayed as a blue mesh contoured at the ≥3.5σ level. Nucleotide-binding sites are shown for the phosphate-bound structure (PDB entry 9i4v), with chain *A* in (*a*) and chain *B* in (*b*), the ADP-bound structure (PDB entry 9i51), with chain *A* in (*c*) and chain *B* in (*d*), and the ATP-bound structure (PDB entry 9i53), with chain *A* in (*e*) and chain *B* in (*f*).

The determined crystal structures revealed that ADP and ATP bind to a site formed by the RecA1 and RecA2 domains of NSP13, as expected, with each nucleotide accompanied by an inorganic phosphate derived from the crystallization conditions (Table 1 ▸). These structures were analysed to identify key interactions between the bound ligands and the two NSP13 molecules (chains *A* and *B*) in the asymmetric unit, as shown in Fig. 2 ▸.

In chain *A* of the ADP-bound structure, ADP establishes multiple interactions within the nucleotide-binding site of NSP13 (Fig. 2 ▸*c*). The purine ring of ADP is sandwiched between the His290 side chain, forming π–π stacking interactions, and Arg442, which engages in cation–π and donor–π interactions, along with a hydrogen bond to Ser264. The ribose moiety interacts with Lys320 via a hydrogen bond, while the α-phosphate forms a hydrogen bond to Gly287. The β-phosphate establishes hydrogen bonds to Gly285, Gly287, Lys288 and Ser289. The inorganic phosphate bound within the nucleotide-binding site forms hydrogen bonds to Gln404 and Gly538. Potential salt bridges are observed between the β-phosphate and Lys288 and Arg443, as well as between the inorganic phosphate and Lys288, Arg443 and Arg567.

**Figure 3:**
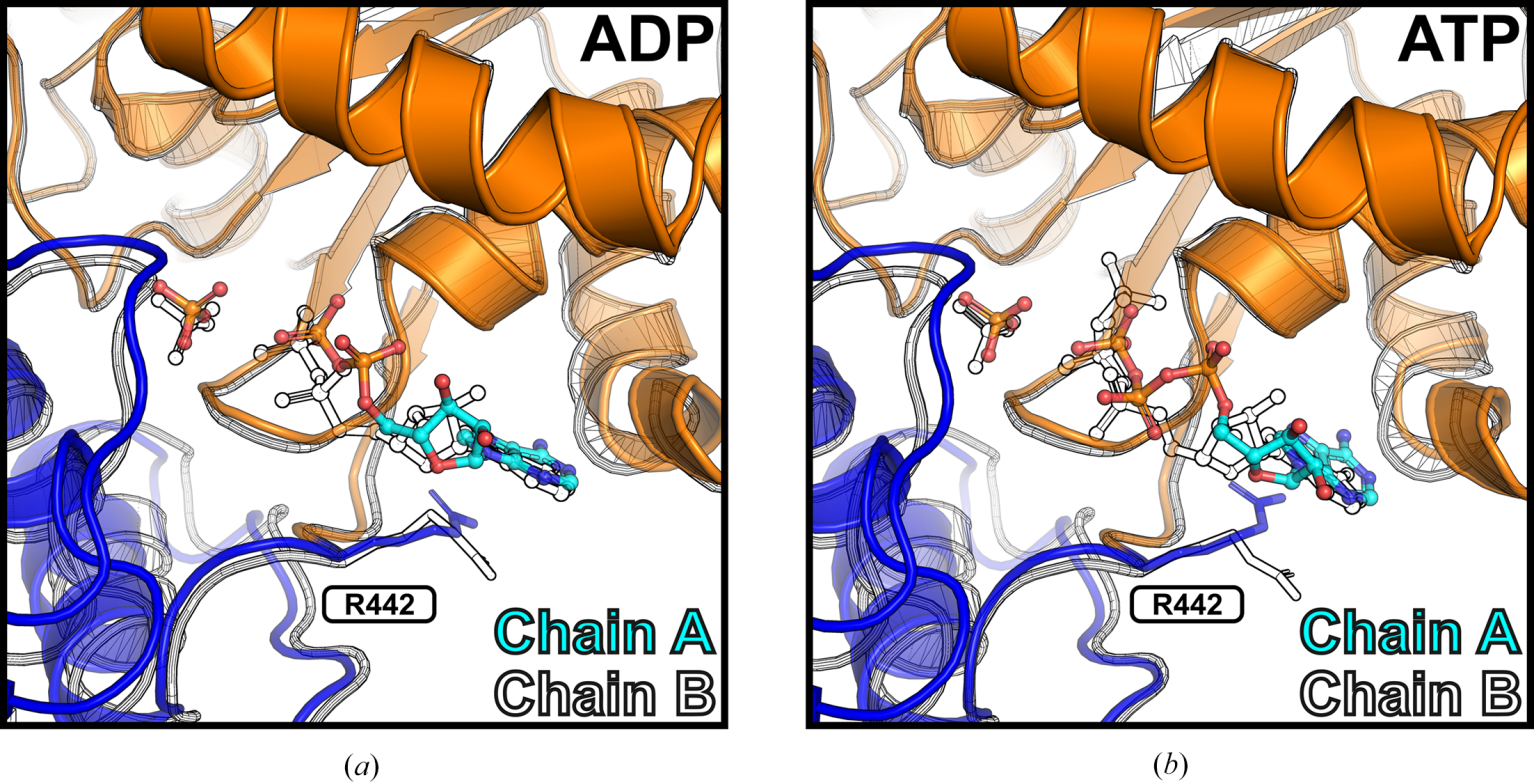
Nucleotide shift between chain *A* and chain *B* of the ADP-bound and ATP-bound structures of SARS-CoV-2 NSP13. NSP13 is shown in cartoon representation, with chain *A* coloured (RecA1 domain in orange, RecA2 domain in blue) and chain *B* outlined in black. The side chains of Arg442 are depicted in stick format, while nucleotides are shown in ball-and-stick format, coloured cyan for chain *A* and outlined in black for chain *B*. Nucleotide shifts between chain *A* and chain *B* are illustrated for the ADP-bound (*a*) and ATP-bound (*b*) structures of NSP13 (PDB entries 9i51 and 9i53, respectively).

Notable differences in ADP binding were observed between chains *A* and *B*. In chain *B* (Fig. 2 ▸*d*), the purine ring of ADP does not form a hydrogen bond to Ser264, as seen in chain *A*. Instead, the ribose forms an additional hydrogen bond to Lys320, and the α-phosphate establishes a potential salt bridge with Arg443. These changes indicate a shift of ADP within the nucleotide-binding site towards the protein atoms of NSP13. In particular, the C1′ atom of the ribose of ADP in chain *B* is displaced by 1.2 Å, while the P atom of the β-phosphate shifts in the same direction by 0.9 Å. The P atom of the inorganic phosphate is shifted by 0.6 Å. Furthermore, the distance between the P atoms of the ADP β-phosphate and the inorganic phosphate decreases by 0.5 Å (distance in chain *A*, 5.0 Å; distance in chain *B*, 4.5 Å). Pairwise structural alignment of the two chains yielded an all-atom r.m.s.d. of 0.99 Å. The nucleotide-binding sites in both chains are nearly identical, with the only significant difference being the conformation of the Arg442 side chain, which undergoes a shift of 3.9 Å (based on the C^ζ^ atoms). This conformational change is likely to trigger the observed nucleotide shift within the binding site of NSP13, as illustrated in Fig. 3 ▸.

In chain *A* of the ATP-bound structure, ATP adopts a binding pattern similar to that of ADP in chain *A* of the ADP-bound structure (Fig. 2 ▸*e*). The purine ring of ATP is sandwiched between His290, forming π–π interactions, and Arg442, establishing cation–π and donor–π interactions, along with a hydrogen bond to Ser264. The α-phosphate of ATP establishes hydrogen bonds to Ser289 and His290, while the ribose forms a hydrogen bond to Lys320. The β-phosphate engages in a hydrogen bond with Gly287, and the γ-phosphate forms hydrogen bonds to Gly285, Gly287, Lys288 and Ser289. The inorganic phosphate observed in the nucleotide-binding site forms hydrogen bonds to Gln404 and Gly538. Potential salt bridges are observed between the α-phosphate and Lys320, the β- and γ-phosphates and Arg443 and Lys288, and the inorganic phosphate and Lys288, Arg443 and Arg567.

Differences in ligand-binding interactions between chains *A* and *B* of the ATP-bound structure closely mirrored those observed in the ADP-bound structure. In chain *B* (Fig. 2 ▸*d*), the purine ring of ATP does not form a hydrogen bond to Ser264, as seen in chain *A*. Instead, the ribose forms hydrogen bonds to Lys320, while the α-phosphate establishes a potential salt bridge with Arg443. The β-phosphate in chain *B* occupies a position similar to the γ-phosphate in chain *A*, while the γ-phosphate is repositioned and forms hydrogen bonds to Lys288 and Ser289. These differences indicate a shift of ATP in chain *B* towards the protein atoms within the nucleotide-binding site of NSP13. This shift is characterized by a 2.0 Å displacement of the C1′ atom of the ribose and a 2.5 Å displacement of the γ-phosphate. The P atom of the inorganic phosphate is shifted by 0.5 Å. Furthermore, the distance between the P atoms of the ATP γ-phosphate and the inorganic phosphate decreases by 0.3 Å (distance in chain *A*, 4.8 Å; distance in chain *B*, 4.5 Å). Pairwise structural alignment of the two chains yielded an all-atom r.m.s.d. of 1.05 Å. The nucleotide-binding sites are highly similar, with the only significant difference being the conformation of the Arg442 side chain, which undergoes a shift of 4.0 Å (based on the C^ζ^ atoms). These structural differences in ATP binding are illustrated in Fig. 3 ▸, aligning with the findings from the ADP-bound structure and underscoring the role of Arg442 in facilitating nucleotide repositioning within NSP13.

**Figure 4:**
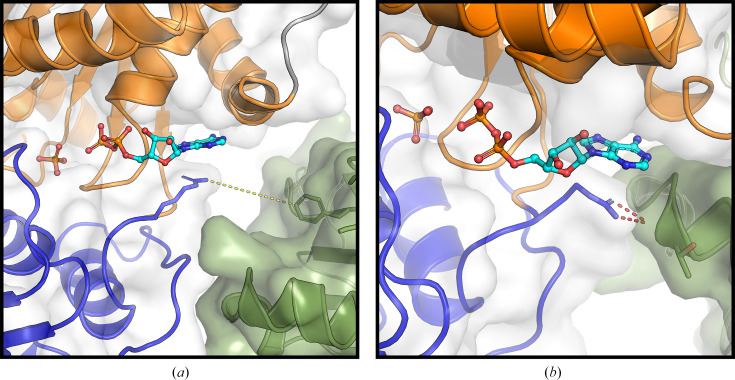
Influence of symmetry mates on the side-chain conformation of Arg442 in the ADP-bound structure of SARS-CoV-2 NSP13. NSP13 is shown in cartoon representation, showing the RecA1 domain (orange) and the RecA2 domain (blue). Bound ADP (cyan) and inorganic phosphate (atom-coloured) are depicted in ball-and-stick format. The symmetry mates are shown in smudge-coloured cartoon format. In chain *A*, the Arg442 side chain is 9 Å from Phe472 of the symmetry mate, indicated by a yellow dashed line (*a*). In chain *B*, the Arg442 side chain forms hydrogen bonds to Thr501 of the symmetry mate, indicated by red dashed lines (*b*). These interactions were also observed in the unliganded, phosphate-bound and ATP-bound structures of SARS-CoV-2 NSP13.

Each nucleotide-bound crystal structure of NSP13 revealed highly similar nucleotide-binding sites in the two chains in the asymmetric unit, differing only in the conformation of Arg442, as shown in Fig. 3 ▸. Furthermore, no differences were observed between corresponding chains of the ADP- and ATP-bound structures, despite the binding of different nucleotides, as shown by an all-atom r.m.s.d. of 0.18 Å for chain *A* and 0.21 Å for chain *B*. These findings prompted an analysis of symmetry mates to assess their potential influence on NSP13 crystal structures. Indeed, symmetry mates were consistently observed in close proximity to the nucleotide-binding site in each chain across all analysed structures, as illustrated in Fig. 4 ▸. In both the ADP- and ATP-bound structures the Arg442 side chain in chain *A* lies within 9 Å of a symmetry mate, while in chain *B* it directly interacts with the main chain of Thr501 of the symmetry mate. These results suggest that symmetry mates near the nucleotide-binding site influence the conformation of Arg442 and may stabilize the nucleotide-binding site of NSP13.

## 4. Discussion

The nucleotide-bound crystal structures of SARS-CoV-2 NSP13 reported here reveal the expected binding of ADP and ATP to the nucleotide-binding site (Fig. 2 ▸). In both cases, the nucleotide binds alongside an inorganic phosphate originating from the crystallization conditions (Table 1 ▸). The nucleotide-binding sites in these structures differed conformationally only in the positioning of the Arg442 side chain, which appears to trigger or accompany the observed nucleotide shift (Fig. 3 ▸). An examination of symmetry mates revealed intermolecular contacts involving Arg442 and the nucleotide-binding site of neighbouring molecules in the crystal lattice (Fig. 4 ▸), potentially stabilizing this site of NSP13.

### 4.1. Inorganic phosphates binding to the nucleotide-binding site of SARS-CoV-2 NSP13

Inorganic phosphates, originating from the crystallization conditions (Table 1 ▸), were consistently observed to bind to NSP13 molecules alongside ADP and ATP (Fig. 2 ▸). Magnesium was deliberately omitted from all soaking solutions, suppressing ATP hydrolysis *in crystallo* and ensuring that the ATP- and ADP-bound data sets were collected under identical metal-free conditions. In the case of the ATP-bound structure (Figs. 2 ▸*e* and 2 ▸*f*), the simultaneous presence of ATP and inorganic phosphate suggests a nonphysiological state, as ATP typically binds only after ADP and orthophosphate (P_i_) have been released during the catalytic cycle (Newman *et al.*, 2021 ▸). In contrast, the ADP-bound structure (Figs. 2 ▸*c* and 2 ▸*d*), which contains both ADP and an inorganic phosphate, mimics a state following ATP hydrolysis in NSP13.

In addition to the structures reported here, inorganic phosphates were observed in the unliganded NSP13 crystals used for soaking (PDB entry 9i4v) and in NSP13 crystals obtained under similar conditions (PDB entry 6zsl). These phosphates occupy positions corresponding to the α- and γ-phosphates in the open product state (Newman *et al.*, 2021 ▸). They form several potential salt bridges, stabilizing their binding in the nucleotide-binding site of NSP13. In particular, the inorganic phosphate at the α-phosphate position may interact with Lys288 and Arg443, while the phosphate at the γ-phosphate position forms potential salt bridges with Lys288, Arg443 and Arg567, potentially resulting in greater stabilization (Figs. 2 ▸*a* and 2 ▸*b*). These interactions are likely to explain why the inorganic phosphate at the α-phosphate position is consistently displaced by ADP or ATP in the reported structures, while the phosphate at the γ-phosphate position remains bound.

**Figure 5:**
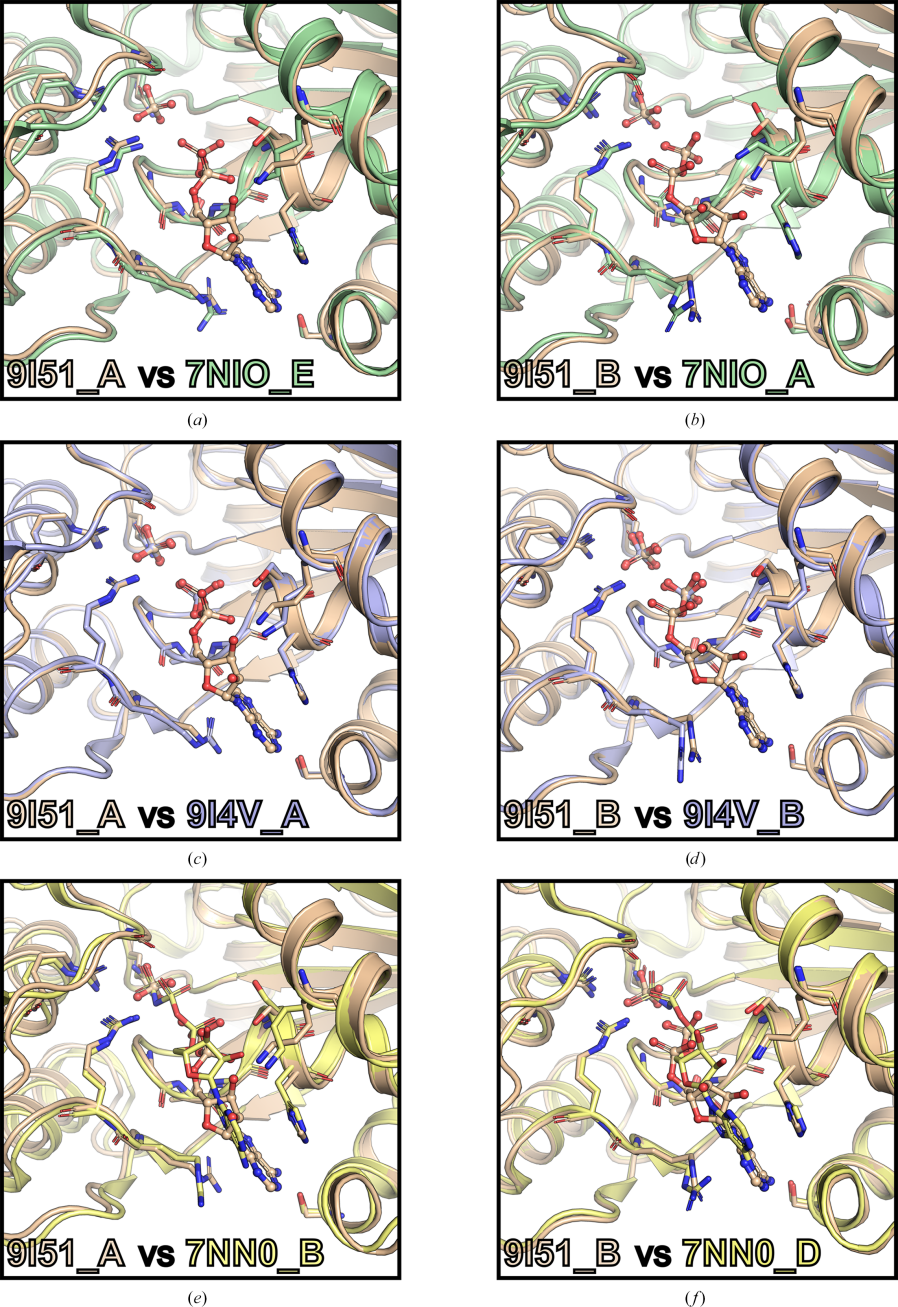
Pairwise structural alignments of each chain of the ADP-bound structure (PDB entry 9i51; wheat) were performed against the corresponding chains of the apo-form (PDB entry 7nio; green), phosphate-bound (PDB entry 9i4v; blue) and AMP-PNP-bound (PDB entry 7nn0; yellow) structures of SARS-CoV-2 NSP13. NSP13 is shown in cartoon format. Nucleotides and phosphates are depicted in ball-and-stick format, coloured to match the respective proteins, while interacting residues are shown in stick format. The following alignments are illustrated: (*a*) chain *A* of the ADP-bound structure with chain *E* of the apo form, (*b*) chain *B* of the ADP-bound structure with chain *A* of the apo form, (*c*) chain *A* of the ADP-bound structure with chain *A* of the phosphate-bound structure, (*d*) chain *B* of the ADP-bound structure with chain *B* of the phosphate-bound structure, (*e*) chain *A* of the ADP-bound structure with chain *B* of the AMP-PNP-bound structure and (*f*) chain *B* of the ADP-bound structure with chain *D* of the AMP-PNP-bound structure.

Pairwise structural comparisons between the phosphate-bound and nucleotide-bound structures yielded all-atom r.m.s.d.s of 0.31 Å for chain *A* and 0.18 Å for chain *B*. Furthermore, the phosphate-bound structures exhibit the same nucleotide-binding site configuration, with the conformation of Arg442 in both chains of the asymmetric unit matching that observed in the ADP-bound and ATP-bound structures (Figs. 5 ▸*c* and 5 ▸*d*). Remarkably, the inorganic phosphate occupying the γ-phosphate position in the phosphate-bound structures aligns with the corresponding inorganic phosphate in the nucleotide-bound structures of NSP13.

### 4.2. The NSP13–ADP complex captures a state immediately following ATP hydrolysis

In order to determine whether the ADP-bound structure reported here corresponds to a state of NSP13 following ATP hydrolysis, this structure was compared with the apo-form structure (PDB entry 7nio) and phosphate-bound structures (PDB entries 6zsl and 9i4v), each representing the open state of NSP13 (Newman *et al.*, 2021 ▸). Pairwise structural comparisons between the ADP-bound structure and the corresponding chains in the apo-form and phosphate-bound structures revealed no significant differences, as shown in Figs. 5 ▸(*a*)–5 ▸(*d*).

Furthermore, the ADP-bound structure was compared with NSP13 co-crystallized with the nonhydrolysable ATP analogue AMP-PNP (PDB entry 7nn0). In the AMP-PNP-bound structure, nucleotides were bound across all four molecules in the asymmetric unit, revealing two distinct binding modes (Newman *et al.*, 2021 ▸). Mode *A*, represented by chain *A*, corresponds to the pre-hydrolysis state and adopts a closed conformation. In contrast, mode *B*, observed in chains *B*, *C* and *D*, adopts an open conformation, reflecting the product state following ATP hydrolysis. Pairwise structural alignments revealed that chain *A* of the ADP-bound structure closely resembles chain *B* of the AMP-PNP-bound structure (all-atom r.m.s.d. of 0.32 Å). Similarly, chain *B* of the ADP-bound structure aligns more closely with chain *D* of the AMP-PNP-bound structure (all-atom r.m.s.d. of 0.30 Å). Both chains represent mode *B*, the open product state of NSP13. Moreover, the nucleotide-binding sites were highly similar, with ADP and AMP-PNP aligning particularly well. The α- and β-phosphates of ADP, as well as the inorganic phosphate at the γ-position, align well with their equivalent positions in AMP-PNP (Figs. 5 ▸*e* and 5 ▸*f*).

Hence, the observed structural similarities between the ADP-bound structure and the product state of the AMP-PNP-bound structure, alongside its resemblance to the apo-form and phosphate-bound structures, provide strong evidence that this conformation represents a nucleotide-bound state immediately following ATP hydrolysis of NSP13.

### 4.3. The role of symmetry mates in stabilizing the nucleotide-binding site of SARS-CoV-2 NSP13

Intriguingly, the ADP- and ATP-bound NSP13 complexes reported here adopt the same overall conformation as the phosphate-bound structure, as all crystals for soaking experiments were grown under identical conditions. The crystal lattice constrains local conformational changes of residues within the nucleotide-binding sites that might otherwise occur upon nucleotide binding. In particular, crystal contacts appear to restrain the conformation of Arg442 in each polypeptide chain, thereby stabilizing the nucleotide-binding site of SARS-CoV-2 NSP13 (Fig. 5 ▸). Interestingly, examination of a structurally related apo-form (phosphate-free) structure (PDB entry 7nio), crystallized in the same *P*1 space group but under different conditions, revealed identical symmetry-mate contacts and similar nucleotide-binding site conformations, especially in the positioning of Arg442 (Figs. 5 ▸*a* and 5 ▸*b*).

In all reported NSP13 structures crystallized in space group *P*1, symmetry mates interact with the nucleotide-binding sites, particularly influencing the conformation of Arg442. These contacts differ between the two monomers in the asymmetric unit, resulting in distinct Arg442 conformations in each NSP13 chain. In the ADP-bound and ATP-bound structures, this variation coincides with shifts in nucleotide positioning, highlighting the potential sensitivity of nucleotide binding to subtle conformational changes in Arg442. Remarkably, beyond Arg442, the nucleotide-binding sites across both chains in all space-group *P*1 structures exhibit identical side-chain conformations, suggesting that crystal packing plays a central role in shaping the nucleotide-binding site of NSP13.

In conclusion, these findings underscore the influence of crystal packing on the study of complex structures obtained through soaking experiments, a well known challenge in crystallographic studies. As demonstrated by the structures reported here, crystal packing can affect the shape of the nucleotide-binding site and complicate the accurate determination of protein–ligand complexes by altering or obstructing the plasticity of binding sites. Furthermore, soaking experiments primarily allow local side-chain rearrangements and cannot resolve the domain-scale conformational changes that may accompany ATP binding and hydrolysis. Such larger-scale movements, which are typical of helicases like NSP13, require co-crystallization or cryo-EM analysis. Indeed, comparison with available cryo-EM structures of NSP13 (Chen *et al.*, 2022 ▸) reveals significant differences in domain positioning, as reflected in elevated r.m.s.d. values. A complete picture of NSP13 dynamics will therefore require integrative structural approaches that capture both local and global conformational changes. Accounting for these effects is essential to ensure the reliability of structure-based drug design and inhibitor development.

## Supplementary Material

PDB reference: SARS-CoV-2 helicase NSP13, complex with ADP, 9i51

PDB reference: complex with ATP, 9i53

## Figures and Tables

**Table 1:** Crystallization

Method	Vapour diffusion
Plate type	Sitting drop
Temperature (K)	293
Protein concentration (mg ml^−1^)	5
Buffer composition of protein solution	12.5 m*M* HEPES pH 7.5, 125 m*M* NaCl, 0.125 m*M* TCEP
Composition of reservoir solution	16% ethylene glycol, 8% PEG 8000, 0.05 *M* HEPES, 0.05 *M* MOPS, 0.03 *M* sodium nitrate, 0.03 *M* sodium phosphate, 0.03 *M* ammonium sulfate, 9% MPD
Volume and ratio of drop	0.5 µl, 1:1 ratio
Volume of reservoir (µl)	40
Composition of cryoprotectant	20% ethylene glycol, 10% PEG 8000, 0.05 *M* HEPES, 0.05 *M* MOPS, 0.03 *M* sodium nitrate, 0.03 *M* sodium phosphate, 0.03 *M* ammonium sulfate, 9% MPD
Seeding type	Microseeding
Volume of seeds (µl)	0.033

**Table 2:** Data collection and processing Values in parentheses are for the outer shell.

Structure	ADP-bound NSP13	ATP-bound NSP13
PDB code	9i51	9i53
Diffraction source	P13, PETRA III, DESY	P13, PETRA III, DESY
Wavelength (Å)	0.9763	0.9763
Temperature (K)	100	100
Detector	EIGER 16M	EIGER 16M
Crystal-to-detector distance (mm)	252	252
Rotation range per image (°)	0.1	0.1
Total rotation range (°)	500	500
Exposure time per image (s)	0.008	0.008
Space group	*P*1	*P*1
*a*, *b*, *c* (Å)	59.31, 70.50, 85.21	59.58, 70.64, 85.89
α, β, γ (°)	103.10, 94.85, 112.74	102.99, 95.29, 112.53
Mosaicity (°)	0.2	0.2
Resolution range (Å)	62.38–1.82 (1.85–1.82)	62.59–1.92 (1.96–1.92)
Total No. of reflections	356268 (13094)	357992 (15428)
No. of unique reflections	100197 (5092)	84816 (4059)
Completeness (%)	91.5 (92.5)	90.3 (87.1)
Multiplicity	3.6 (2.6)	4.2 (3.8)
〈*I*/σ(*I*)〉	6.1 (2.1)	10.8 (2.3)
*R*_meas_	0.062 (0.525)	0.075 (0.667)
Overall *B* factor from Wilson plot (Å^2^)	30.5	29.8

**Table 3:** Structure solution and refinement Values in parentheses are for the outer shell.

Structure	ADP-bound NSP13	ATP-bound NSP13
PDB code	9i51	9i53
Resolution range (Å)	34.22–1.82 (1.84–1.82)	31.81–1.92 (1.94–1.92)
No. of reflections, working set	100191 (3310)	84801 (3028)
No. of reflections, test set	5022 (160)	4245 (142)
Final *R*_cryst_	0.17 (0.32)	0.19 (0.30)
Final *R*_free_	0.20 (0.32)	0.23 (0.33)
No. of non-H atoms		
Total	9570	9596
Protein	8970	8991
Ligand	128	140
Water	472	465
Protein residues	1164	1168
R.m.s. deviations		
Bond lengths (Å)	0.006	0.006
Angles (°)	0.84	0.85
Average *B* factors (Å^2^)		
Overall	43.77	42.42
Protein	43.57	42.30
Ligand	54.71	52.20
Water	44.61	41.83
Ramachandran plot		
Most favoured (%)	97.04	96.19
Allowed (%)	2.78	3.72
Outliers (%)	0.09	0.09
Rotamer outliers (%)	0.91	1.21
Clashscore	2.62	3.72
